# Impact of water ion content on milk protein concentrate gel properties

**DOI:** 10.3389/fnut.2025.1764485

**Published:** 2026-01-12

**Authors:** Yanbing Xu, Xilong Zhou, Junhui Diao, Juan Zhang, Zhenmin Liu

**Affiliations:** 1 School of Life Sciences, Shanghai University, Shanghai, China; 2 Key Laboratory of Dairy Biotechnology, Shanghai Engineering Research Center of Dairy Biotechnology, Dairy Research Institute, Bright Dairy & Food Co., Ltd., Shanghai, China; 3 College of Food Science and Technology, Shanghai Ocean University, Shanghai, China

**Keywords:** boiled tap water, gel, ions, large amplitude oscillatory shear (LAOS), milk protein concentrate, reverse osmosis water, rheology

## Abstract

This study investigated how differences in trace ions present in food industrial and household water affect the formation and properties of milk protein concentrate (MPC) gels. MPC dispersions were prepared using reverse osmosis (RO) water and boiled tap water, a type of water commonly used for daily household consumption, while dispersions prepared with ultrapure water served as the control. The regulatory role of trace ions was systematically characterized by analyzing the ionic composition of the water, the conductivity and zeta potential of the MPC dispersions, the microstructure, water holding capacity, and rheological properties of the MPC gels. The results show that even modest ion content (1.4 mmol/L) in boiled tap water can significantly alter MPC gel properties. Although gels prepared with boiled tap water exhibited a denser microstructure, their elastic modulus and water holding capacity were lower than those of gels prepared with RO water. These outcomes are attributed to the higher concentrations of divalent cations (Ca^2+^ and Mg^2+^) in boiled tap water, which induced pre-aggregation through electrostatic screening and salt-bridge formation, ultimately leading to insufficient cross-linking between aggregates. Nonetheless, due to the low ion content in RO water and boiled tap water, the resulting gels showed no differences in appearance and rheological behavior under large deformation.

## 1 Introduction

Milk protein concentrate (MPC) powder is a dairy derived ingredient from skim milk through ultrafiltration, vacuum concentration, and spray drying (1). Owing to its high protein content and low concentrations of fat and lactose, MPC is widely used in food manufacturing. It serves not only as a protein fortifier to enhance the protein content and nutritional value of cheese and formulated nutritional foods, but also as a fat replacer to improve the mouthfeel and texture of ice cream (2, 3). In yogurt production, the addition of MPC can increase protein content of final products, reduce syneresis, and promote the formation of a denser microstructure, thereby improving texture and stability (4–6). Accordingly, understanding the mechanisms underlying MPC gel formation and the factors regulating its gelation is of great importance for maximizing its functional performance.

Research on MPC gel properties has primarily focused on processing conditions, the production and rehydration of MPC powders, and various gelation-inducing methods (6–11). Processing factors, such as preheating treatment, pH, and incubation temperature, have significant effects on MPC gel formation (7, 8, 12). For example, different preheating intensities lead to varying degrees of protein denaturation, which alter hydrophobic interactions and disulfide bond formation, ultimately affecting the structure and properties of the final gel network (8). The production process of MPC and their rehydration behavior are also critical for the gelation properties of MPC: spray drying and rehydration influence protein solubility and aggregation state, thereby affecting final gel structure and strength (8). In addition, different gelation-inducing methods, such as acid-induced, enzyme-induced, and high-pressure-induced gelation have distinct gelation mechanisms, result in gels with varied microstructures and textural characteristics (6, 10, 11, 13).

Beyond these factors, ions also play an important role in dairy protein gelation. It is widely accepted that ions regulate protein aggregation and gel network formation through electrostatic screening and ionic bridging, with specific effects depending on the species and concentration of the ions (14–17). Both sodium and calcium ions enhance the gelation properties of dairy proteins. In acid-induced milk gels, Kamal et al. (18) reported that the addition of 10 mmol/L CaCl₂ reduced the gelation time from 26.375 min to 7.25 min and increased the final elastic modulus from 20.28 Pa to 222.65 Pa. Similarly, Barbut and Drake (14) found that increasing the sodium ion concentration from 75 to 400 mmol/L significantly enhanced the strength and water holding capacity of whey protein cold-set gels. The strengthening effect of calcium ions is more pronounced than that of sodium ions. For example, heat-induced β-lactoglobulin gels containing 10 mmol/L CaCl₂ showed much higher gel strength than those containing the same concentration of NaCl (15). This difference arises from their distinct mechanisms: sodium ions predominantly screen electrostatic repulsion between protein molecules, promoting hydrophobic interactions and protein aggregation (16), whereas calcium ions exhibit more complex regulatory effects. In addition to electrostatic screening, calcium ions form “calcium bridges” with phosphate and carboxyl groups on casein molecules, thereby promoting intermolecular cross-linking. Calcium ions may also bind phosphate ions, altering ionic equilibrium, releasing H^+^, and decreasing pH slightly, which further affects gel structure and properties. Although these ions promote gelation at moderate concentrations, excessive ionic strength can impair gel properties. For instance, when sodium ion concentrations exceed 400 mmol/L or calcium ion concentrations exceed 10 mmol/L, the gel strength and water holding capacity of heat-induced β-lactoglobulin gels decrease sharply (15). Similarly, sodium ion concentration exceeding 200 mmol/L reduces the water holding capacity and strength of heat-induced whey protein gels (16).

Most existing studies evaluated ionic effects on gel properties by adding NaCl or CaCl₂ at concentrations typically exceeding 5 mmol/L that is much higher than the actual ionic concentrations (<2 mmol/L) present in reverse osmosis (RO) water used in industrial food production or in tap water commonly consumed by the public (19–21). Although monovalent and divalent cations could theoretically affect protein aggregation, gel network formation, and the resulting functional properties, their effects in a trace concentration range, such as those naturally present in water, remains insufficiently explored. Assessing how water sources influence MPC gelation and gel properties is therefore essential, not only for bridging the gap between laboratory conditions, industrial production and consumer use, but also for establishing a scientific basis for controlling process consistency and ensuring product uniformity.

In this study, MPC dispersions prepared using RO water and boiled tap water were employed as model systems to systematically evaluate how trace ions regulate intermolecular interactions, gel microstructure, and the rheological behavior of milk protein gels. Ultrapure water, from which trace ions are effectively eliminated, was used as a control to verify the role of trace ions. Furthermore, the mechanisms by which differences in water affect the structure and mechanical properties of MPC gels were analyzed from a multiscale perspective. In addition to conventional time sweep rheological measurements, large amplitude oscillatory shear tests were employed to quantify the nonlinear rheological characteristics of gels.

## 2 Materials and methods

### 2.1 Material

Glucono-δ-lactone (GDL, purity ≥ 99%) was purchased from Merck (China). Cyanine 5 NHS ester dye was obtained from Lumiprobe (USA). GeneFrame (17 × 28 mm) was purchased from Thermo Fisher Scientific (China). Coverslips (24 × 40 mm, thickness 0.13–0.17 mm) were purchased from Sangon Biotech Co., Ltd. (China). Membrane filters (cutoff 0.45 μm, PES) were purchased from Aladdin (China).

The MPC powder was produced in the pilot plant of the Bright Dairy & Food Co., Ltd. East China Central Factory. According to the analysis carried out by SGS-CSTC Standards Technical Services Co., Ltd. (China), the MPC powder contained 84.1 wt% protein (62.8 wt% casein and 16.3 wt% whey protein), 2.6 wt% fat, 0.8 wt% lactose, 0.2 wt% calcium, and 6.5 wt% ash.

Ultrapure water was prepared using a Milli-Q system. RO water was provided by the East China Central Factory of Bright Dairy & Food Co., Ltd. Boiled tap water was prepared by boiling municipal tap water from Shanghai, China, cooling it, and subsequently filtering it through a 0.45 μm PES membrane filter. The concentrations of cations in the above water samples were analyzed by SGS-CSTC Standards Technical Services Co., Ltd. (China). The quantitation limits were 0.06 mg/L for sodium ions, 0.16 mg/L for potassium ions, 1.2 mg/L for magnesium ions, and 1.7 mg/L for calcium ions.

### 2.2 Preparation of acid-induced gels

The MPC powder was dissolved in the three types of water used in this study reaching a protein concentration of 4 wt%. The MPC dispersions were hydrated at room temperature for 3 h, followed by an additional 1 h of hydration at 50 °C to improve MPC solubility. The dispersions were then preheated at 99 °C for 10 min in a thermostatic water bath while being magnetically stirred at 200 rpm. Immediately after preheating, the samples were cooled to room temperature using an ice-water bath. The conductivity of the samples was measured using a DDS-307A conductivity meter (Shanghai INESA Scientific Instrument Co., Ltd., China). MPC dispersions prepared using ultrapure water, RO water, and boiled tap water were designated as MPC (UPW), MPC (ROW), and MPC (BTW), respectively.

Glucono-δ-lactone (GDL) was then added to the MPC dispersions to induce gelation. The mass ratio of GDL to protein was 1:4, corresponding to 1 wt% in the dispersions. The samples were gently stirred until the GDL was completely dissolved and subsequently transferred to a 40 °C thermostatic water bath for static incubation for 3 h to complete the gelation process.

### 2.3 Particle size

The particle size distribution of proteins in the MPC dispersions was measured using a ZetaSizer Nano-ZS (Malvern Instruments Ltd., United Kingdom). Each MPC dispersions were diluted with the same solvent used for their preparation (ultrapure water, RO water, or boiled tap water) to a final protein concentration of 0.1 wt%. Then, 1 mL of a diluted sample was transferred into a disposable cuvette (DTS0012). The refractive index and absorption coefficient of proteins were set to 1.450 and 0.001, respectively, and the refractive index of the solvent was set to 1.330. All samples were equilibrated at 25 °C for 30 s prior to measurement.

### 2.4 Zeta potential

The zeta potential of proteins in the MPC dispersions was determined using a ZetaSizer Nano-ZS (Malvern Instruments Ltd., United Kingdom). The MPC dispersions were diluted with the same type of water used in their preparation (ultrapure water, RO water, or boiled tap water) to a protein concentration of 0.1 wt%. Then, 1 mL of the diluted sample was transferred into a zeta potential cell (DTS1070) for measurement.

### 2.5 Dynamic pH change during gelation

The pH evolution during gelation was monitored using an iCinac dairy fermentation analyzer (Alliance Instruments, France). A volume of 20 mL of MPC dispersion was placed in a 50 mL centrifuge tube, followed by the addition of 0.2 g GDL. After complete dissolution via vortex mixing, the tube was incubated in a 40 °C water bath. The pH electrode was inserted into the middle of the liquid, and pH was continuously monitored over 3 h.

### 2.6 Water holding capacity (WHC)

The WHC of the gels was determined following the method of Urbonaite et al. (2016) (22), with minor modifications. A 10 g of MPC dispersion was transferred into a 15 mL centrifuge tube and gelled according to the method described in Section 2.2. Samples were centrifuged at 4 °C at 400 × g for 15 min. After centrifugation, the supernatant was carefully removed and weighed. WHC was calculated using the following equation:


$$\mathrm{WHC}=\frac{m_{\mathrm{gel}}\times 96\%-m_{\text{serum}}}{m_{\mathrm{gel}}\times 96\%}$$

where $m_{\text{serum}}$ is the mass of the supernatant, $m_{\mathrm{gel}}$ is the initial gel mass before centrifugation, and 96% represents the initial moisture content of the gel.

### 2.7 Microstructure

The microstructure of the MPC gels was observed using a confocal laser scanning microscope. Sample preparation followed the method described by Zhou et al. (8). Briefly, 1 mL of preheated MPC dispersion was mixed with 50 μL of Cyanine 5 NHS ester dye solution (1 mg/mL in DMSO) for staining. Then, 0.01 g GDL was added to the stained dispersion. After complete dissolution of GDL, the mixture was transferred into a custom-made sample chamber (constructed by adhering a GeneFrame to a coverslip), sealed with a plastic film, wrapped in aluminum foil to prevent light exposure, and incubated at 40 °C for 3 h to allow gel formation. After incubation, the samples were cooled to 4 °C and examined using a STELLARIS 5 confocal microscope (Leica Microsystems, Germany). The excitation wavelength was set to 638 nm, and fluorescence emission was collected from 647 to 746 nm.

### 2.8 Rheological properties

The dynamic gelation behavior of MPC dispersions was monitored using an MCR 302E rheometer (Anton Paar, Austria) by recording changes in elastic modulus (G′) and loss modulus (G″) over time. The measurement procedure followed Zhou et al. (8). A 4.7 mL aliquot of the preheated MPC dispersion containing GDL was loaded into a C-CC17/S concentric cylinder geometry. The sample was subjected to an oscillatory shear test at 40 °C for 3 h, with a strain amplitude of 0.1% and frequency of 0.1 Hz. The G’ and G” were recorded every 5 min.

After the time sweep, the sample was cooled to 4 °C at a rate of 1 °C/min and held for 5 min. Subsequently, at 4 °C and a shear frequency of 0.1 Hz, large amplitude oscillatory shear (LAOS) tests were conducted with strain amplitudes logarithmically increased from 0.1% to 100%. G′, G″, and the raw waveform data were recorded. Stress (*σ*) and strain (*γ*) values extracted from the raw waveform were used to generate Lissajous curves (σ vs. γ).

### 2.9 Statistical analysis

All experiments were conducted in three independent replicates, except for the microstructure observations. Each measurement was performed at least twice. Data are presented as mean ± standard deviation. Differences among treatment groups were evaluated using one-way analysis of variance (ANOVA), followed by a Tukey’s post-hoc test for multiple comparisons. Statistical significance was defined as *p* < 0.05.

## 3 Results and discussion

### 3.1 Ion content in water

As shown in Table 1, the total concentration of monovalent ions (Na^+^ and K^+^) in boiled tap water was 1.02 mmol/L, with sodium ions being predominant at 0.94 mmol/L. Among the divalent ions, the concentrations of calcium and magnesium ions were 0.94 mmol/L and 0.42 mmol/L, respectively, giving a total of 1.36 mmol/L, that is higher than that of the monovalent ions.

**Table 1:** Cation content of different water samples.

Sample	The content of sodium ions (mmol/L)	The content of potassium ions (mmol/L)	The content of magnesium ions (mmol/L)	The content of calcium ions (mmol/L)
Ultrapure water	0.00 ± 0.00^a^	0.00 ± 0.00^a^	0.00 ± 0.00^a^	0.00 ± 0.00^a^
Reverse osmosis water	0.41 ± 0.00^b^	0.03 ± 0.00^b^	0.00 ± 0.00^a^	0.07 ± 0.00^b^
Boiled tap water	0.94 ± 0.00^c^	0.08 ± 0.00^c^	0.42 ± 0.00^b^	0.94 ± 0.00^c^

Different letters within the same column indicate significant differences (*p* < 0.05). Values reported as ‘0.00 ± 0.00’ indicate concentrations below the analytical method’s quantitation limit and therefore not quantifiable.

Compared with boiled tap water, RO water contained significantly lower divalent ionic concentration due to the removal of most dissolved minerals during reverse osmosis. As indicated in Table 1, the total concentration of divalent ions (Ca^2+^ and Mg^2+^) in boiled tap water is approximately 19 times higher than that in RO water. The total concentration of monovalent ions in RO water was 0.44 mmol/L, roughly half that of boiled tap water, although the difference was far less pronounced than that observed for divalent ions.

Ultrapure water undergoes multiple purification steps, including ion-exchange adsorption, activated carbon adsorption, reverse osmosis, and UV irradiation, which effectively eliminate major cations such as sodium, potassium, magnesium, and calcium (23). As shown in Table 1, the ion concentrations were all below the quantification limit.

### 3.2 MPC dispersions

#### 3.2.1 Electrical conductivity

Electrical conductivity reflects the ability of a liquid to conduct electricity and is primarily determined by the concentration of dissolved ions, ionic strength, and measurement temperature (24). Therefore, measuring the electrical conductivity of water or protein dispersions provides a practical proxy for the total content of mobile ions, particularly when direct quantification of ionic species in complex protein dispersions is challenging.

As shown in Table 2, the conductivity of the water samples corresponds closely with their cation concentrations: boiled tap water exhibits the highest ionic concentration and thus the highest conductivity, followed by RO water, whereas ultrapure water shows the lowest conductivity.

**Table 2:** Electrical conductivity of different water samples and their corresponding MPC dispersions.

Sample	Electrical conductivity (μs/cm)		
	Ultrapure water	Reverse osmosis water	Boiled tap water
Water samples	0.00 ± 0.00^a^	71.77 ± 1.50^b^	375.00 ± 4.44^c^
MPC dispersions	517.50 ± 7.00^d^	550.00 ± 2.78^e^	641.67 ± 10.07^f^

Different letters indicate significant differences (*p* < 0.05).

The electrical conductivity of MPC dispersions was significantly higher than that of the water used for their preparation. This increase arises because MPC powder releases soluble mineral ions (e.g., calcium and phosphate) during dissolution, and protein molecules disperse to expose charged groups such as carboxyl and amino groups. Together, these factors increase the number of mobile ions in the solution, thereby markedly enhancing its conductivity (25).

When MPC dispersions were prepared with low-conductivity water (ultrapure water or RO water), the resulting dispersions exhibited a substantial increase in conductivity compared with the corresponding water, rising from 71.77 μS/cm to 550.00 μS/cm in RO system and from 0.00 μS/cm to 517.50 μS/cm in ultrapure water system. In contrast, when using boiled tap water, which already had high ionic strength, the conductivity increased only modestly, from 375.00 μS/cm to approximately 641.67 μS/cm. This phenomenon may be attributed to the higher initial ionic strength of boiled tap water, particularly its elevated Ca^2+^ and Mg^2+^ concentrations. These divalent cations stabilized calcium and magnesium in bound form, primarily as colloidal calcium phosphate within casein micelles, thereby inhibiting the dissociation of micellar calcium and reducing the release of free calcium and phosphate into the continuous phase (26, 27). Consequently, the magnitude of the conductivity increase in the dispersion was limited.

Nevertheless, the conductivity of the prepared MPC dispersions still followed the order MPC (BTW) > MPC (ROW) > MPC (UPW). This result indicates that, although milk proteins have a certain ion-buffering effect, the water used for dispersion still significantly influenced the free ion content of the final system. In general, the higher the ionic content of the water, the higher the free ion concentration in the MPC dispersion.

#### 3.2.2 Zeta potential

In milk protein systems, intermolecular electrostatic interactions are key determinants of dispersion stability and gelation behavior and can be quantified with zeta potential. Zeta potential is influenced not only by the ionizable amino acid residues on protein surfaces but also by the ionic composition of the surrounding medium (28–31). Different ion types and concentrations modulate the zeta potential through distinct mechanisms. Monovalent ions, such as sodium, primarily increase the ionic strength, compress the electrical double layer, and thereby reduce the absolute zeta potential (32). In contrast, divalent cations, such as Ca^2+^ and Mg^2+^, exert more complex effects: in addition to electrostatic screening, they can participate in charge neutralization and induce pH reduction. Specifically, the introduction of Ca^2+^ or Mg^2+^ can promote precipitation of phosphate species (e.g., calcium phosphate or magnesium phosphate), releasing hydrogen ions, lowering the pH, and further reducing double-layer thickness (33, 34).

As shown in Table 3, the zeta potentials of MPC (ROW) and MPC (UPW) did not differ significantly, whereas MPC (BTW) exhibited a markedly lower absolute zeta potential (−18.68 ± 0.86 mV), indicating weakened surface negative charge and reduced electrostatic repulsion between protein molecules. Differences in ionic composition between RO water and ultrapure water were mainly due to monovalent cations, which did not result in significant changes in the zeta potential of the corresponding MPC dispersions. In contrast, boiled tap water differed substantially from RO water in divalent cation content, and the significant difference in zeta potential between MPC (BTW) and MPC (ROW). The results suggest that the reduced zeta potential in MPC (BTW) was attributable to the divalent cations (Ca^2+^ and Mg^2+^) present in boiled tap water.

**Table 3:** Zeta potential of MPC dispersions prepared with different water samples.

Sample	Zeta potential (mV)
MPC (UPW)	−27.42 ± 0.46^a^
MPC (ROW)	−26.3 ± 0.84^a^
MPC (BTW)	−18.68 ± 0.86^b^

Different letters indicate significant differences (*p* < 0.05).

#### 3.2.3 Particle size

Previous studies have shown that ionic strength can strongly influence protein aggregation and particle size. For instance, Wu et al. (35) reported that increasing NaCl concentration from 50 to 80 mM induced aggregation of β-casein monomers (~10 nm) into clusters of ~300 nm. This was attributed to the reduction in protein surface charge with increasing NaCl, which weakened electrostatic repulsion, thereby promoting protein association. Similarly, Müller-Buschbaum et al. (36) found that increasing the calcium ion concentration from 10 to 110 mM enlarged the radius of casein particles from 140 nm to 315 nm, indicating that cations promote protein aggregation and increase particle size.

**Figure 1:**
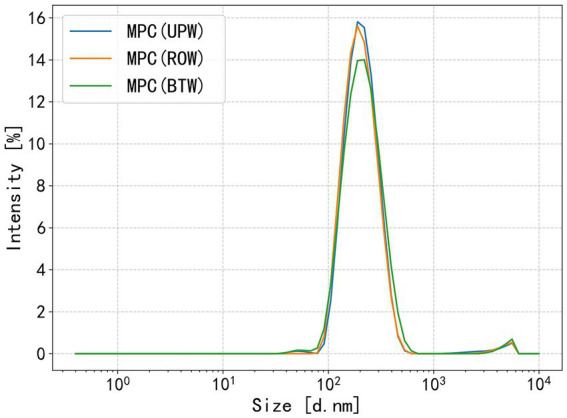
Particle size of MPC dispersions prepared with different water.

In the present study, particle size measurements (Figure 1) revealed no significant differences among MPC dispersions. All dispersions showed peak particle sizes of approximately 200 nm, consistent with the typical size of casein micelles (37). This lack of variation may be explained by the relatively small differences in ion concentrations among the water samples, which were insufficient to induce noticeable protein aggregation under the experimental conditions used.

### 3.3 Gelation process of MPC

Changes in the ionic environment can markedly influence the acid–base balance of milk protein dispersions. Previous studies have reported that adding 600 mM NaCl to concentrated milk reduces the pH from 6.55 to 6.27, primarily because increased ionic strength promotes partial dissociation of ion pairs and the release of bound protons, thereby raising the concentration of free hydrogen ions and causing a measurable pH decrease (32). Sodium ions may also bind to negatively charged groups on caseins, such as phosphoserine residues, displacing protons and further increasing the hydrogen ion concentration (38). Similarly, the addition of calcium chloride lowers the pH of milk. This occurs because exogenous calcium disturbs the intrinsic calcium–phosphate equilibrium and the associated acid–base balance. Added Ca^2+^ shifts the equilibrium between colloidal calcium phosphate and free calcium/phosphate toward reactions that release protons, thereby increasing the concentration of free hydrogen ion. Together, these effects elevate the hydrogen ion concentration in the system, resulting in a pH decrease (39).

**Figure 2:**
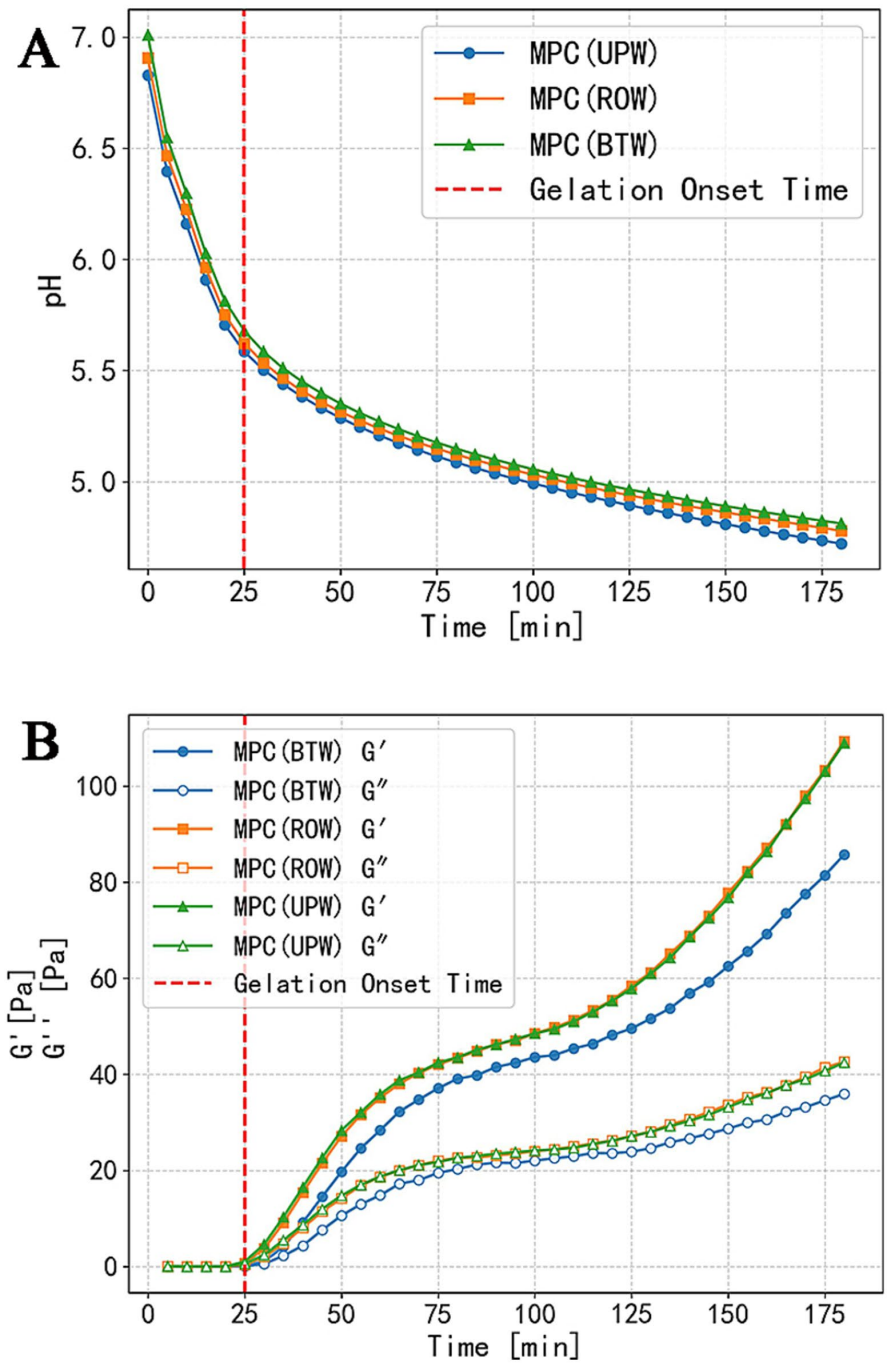
Effects of different water on the gelation process of MPC dispersions: pH variation **(A)**; evolution of elastic (G’) and viscous (G”) modulus **(B)**. The red dashed line indicates the gelation onset time.

However, dynamic pH monitoring during gelation (Figure 2A) indicated that the gelation onset of MPC dispersions prepared with different water consistently occurred at pH 5.6, with nearly identical pH track throughout the gelation process. These results indicate that, under the conditions of this study, the ion concentration differences among the water samples were insufficient to produce measurable changes in the acid–base equilibrium of the system.

In addition to modifying acid–base equilibria, divalent cations can coordinate simultaneously with multiple negatively charged functional groups on protein molecules (e.g., carboxyl groups). This enables them to form “salt bridges” between protein molecules, increasing crosslink density, and facilitating the formation of a three-dimensional protein network (10).

The rheological results (Figure 2B) indicate that gels prepared with different water reached the gelation onset time (G’ > 0.1 Pa) at the same time. Following gelation onset, all samples exhibited the same characteristic pattern: a rapid increase in G’, followed by a slower rise, and then a second rapid increase. During the initial stage, G’ rose sharply and exceeded the loss modulus G,” indicating a well-defined solution–gel transition in which particles or small aggregates rapidly associated to form a system-spanning network structure (40). The subsequent slowdown in G’ development corresponds to internal rearrangements and structural optimization within the nascent gel network. In the later stage, with further crosslinking and densification of the network, as well as continued formation of physical bonds (hydrophobic interactions and calcium bridges) and chemical bonds (disulfide bonds), the gel structure strengthened and G’ increased rapidly once again (41). The final G’ values of MPC (ROW) gel and MPC (UPW) gel reached 109 Pa, both higher than that of MPC (BTW) gel, whose G’ was 85 Pa. Further analysis is provided in the following sections.

### 3.4 Gel properties of MPC gels

#### 3.4.1 Gel appearance

**Figure 3:**
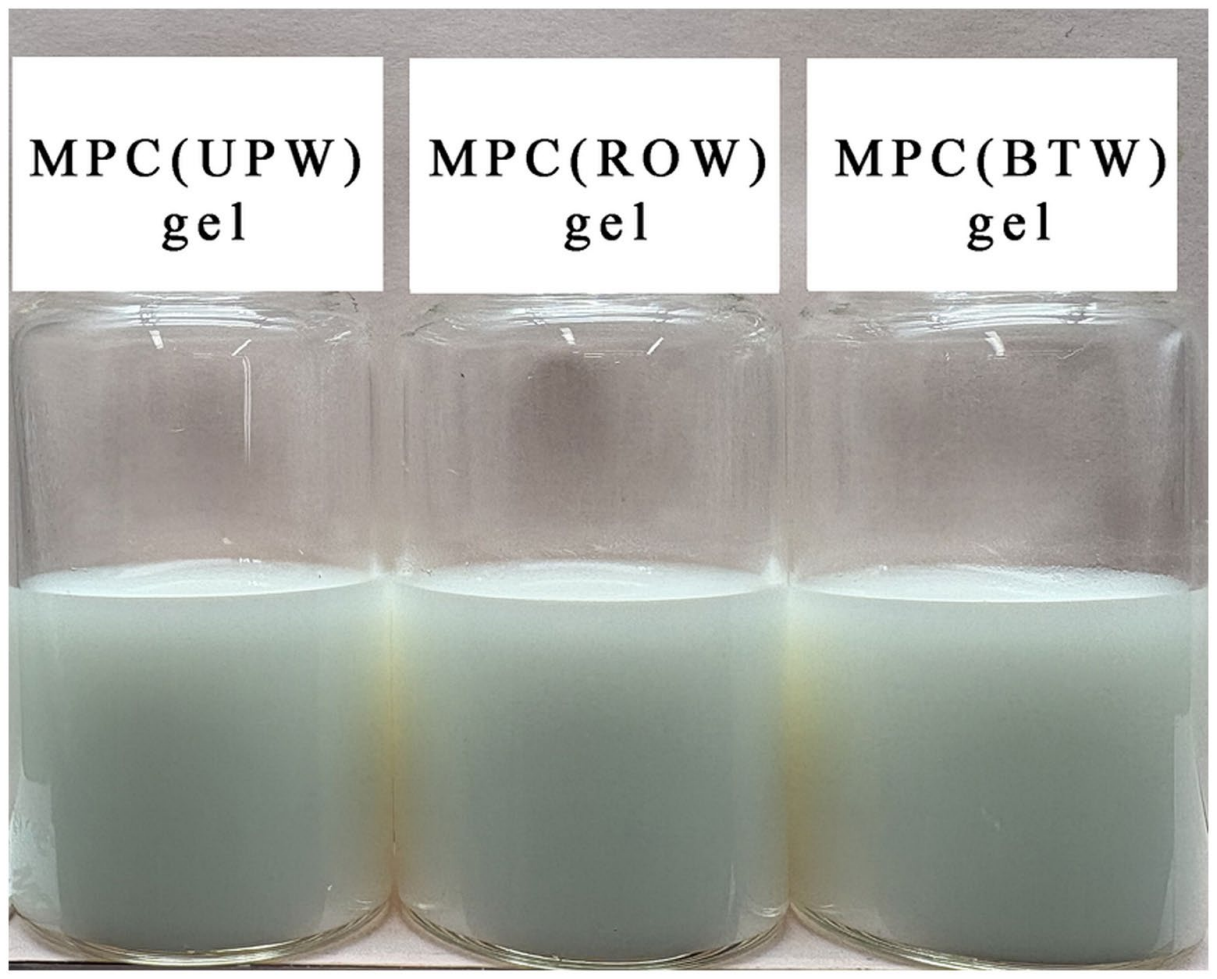
Appearance of gels prepared from MPC dispersions using different types of water.

As shown in Figure 3, gels prepared using different water all exhibited smooth and uniform surfaces without syneresis or visible collapse. No macroscopic differences were observed among the samples, indicating that the variations in ionic concentration among RO water, boiled tap water, and ultrapure water had no noticeable influence on gel appearance.

#### 3.4.2 Water holding capacity and microstructure

**Figure 4:**
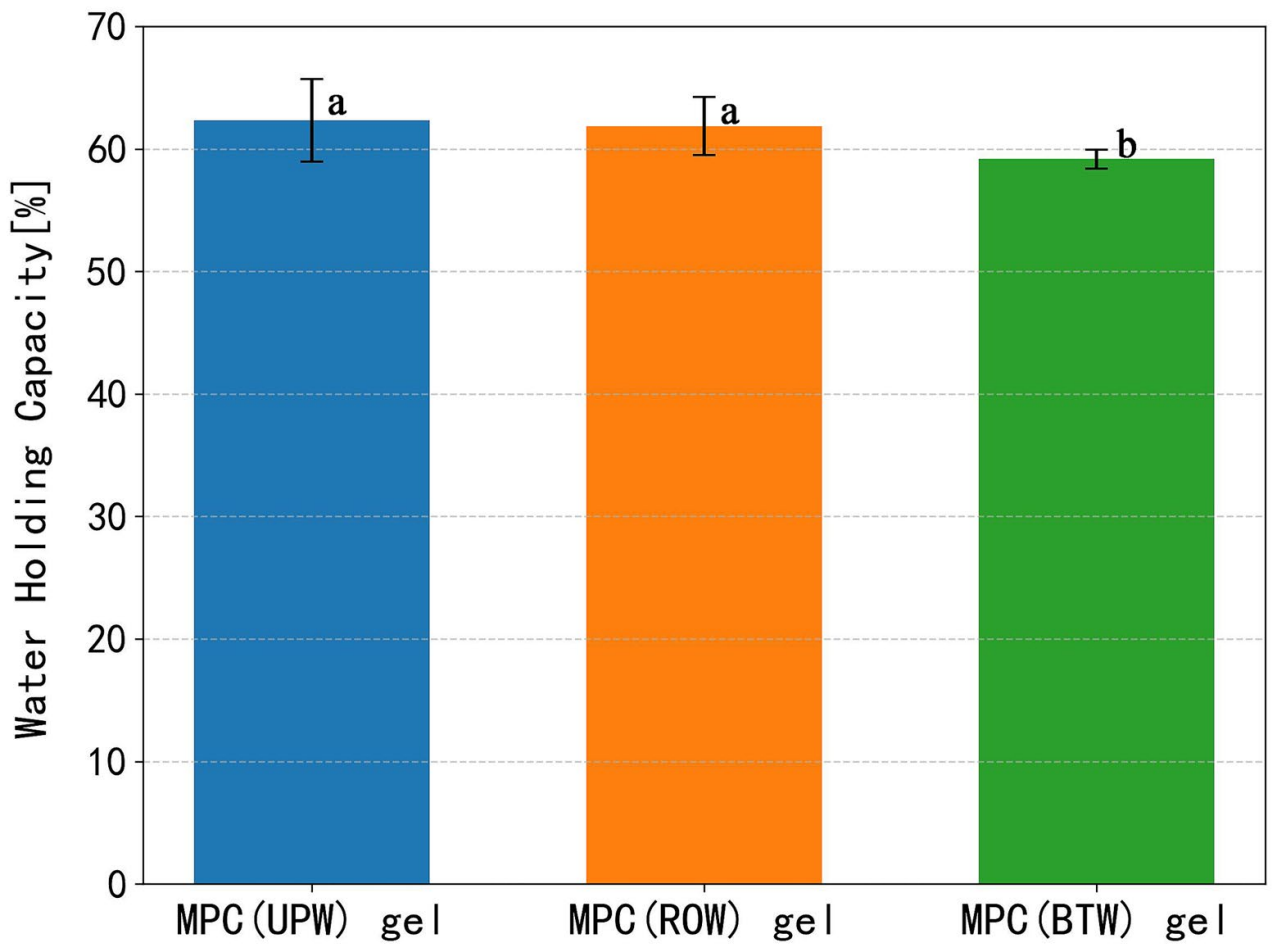
Water holding capacity of MPC gels prepared with different water. Gels were centrifuged at 400 × *g* for 15 min. Different letters indicate significant differences between groups (*p* < 0.05).

The WHC is a key indicator of the moisture-retention ability of dairy protein gels and is closely associated with its texture, mouthfeel, and stability. As shown in Figure 4, MPC (ROW) gel and MPC (UPW) gel exhibited comparable WHC, both significantly higher than that of MPC (BTW) gel. The WHC of protein gels is strongly influenced by their network structure (42).

**Figure 5:**
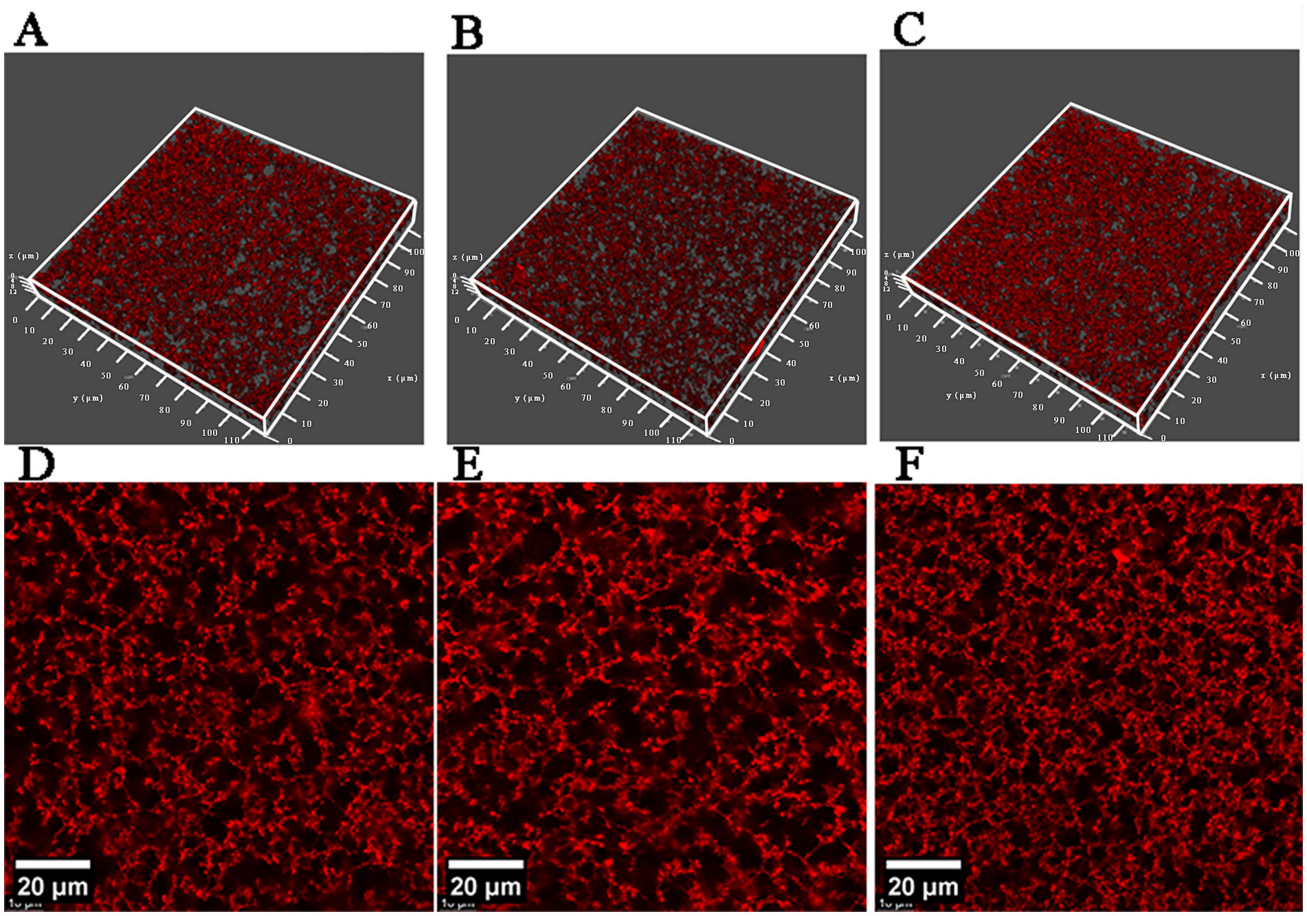
Microstructure of gels prepared from MPC dispersions using different types of water. Three-dimensional images of gels obtained by laser confocal microscopy (size: 114 μm × 114 μm × 15 μm) **(A–C)**; two-dimensional planar images of the gels obtained by laser confocal microscopy **(D–F)**. MPC (UPW) gel **(A,D)**; MPC (ROW) gel **(B,E)**; MPC (BTW) gel **(C,F)**.

**Figure 6:**
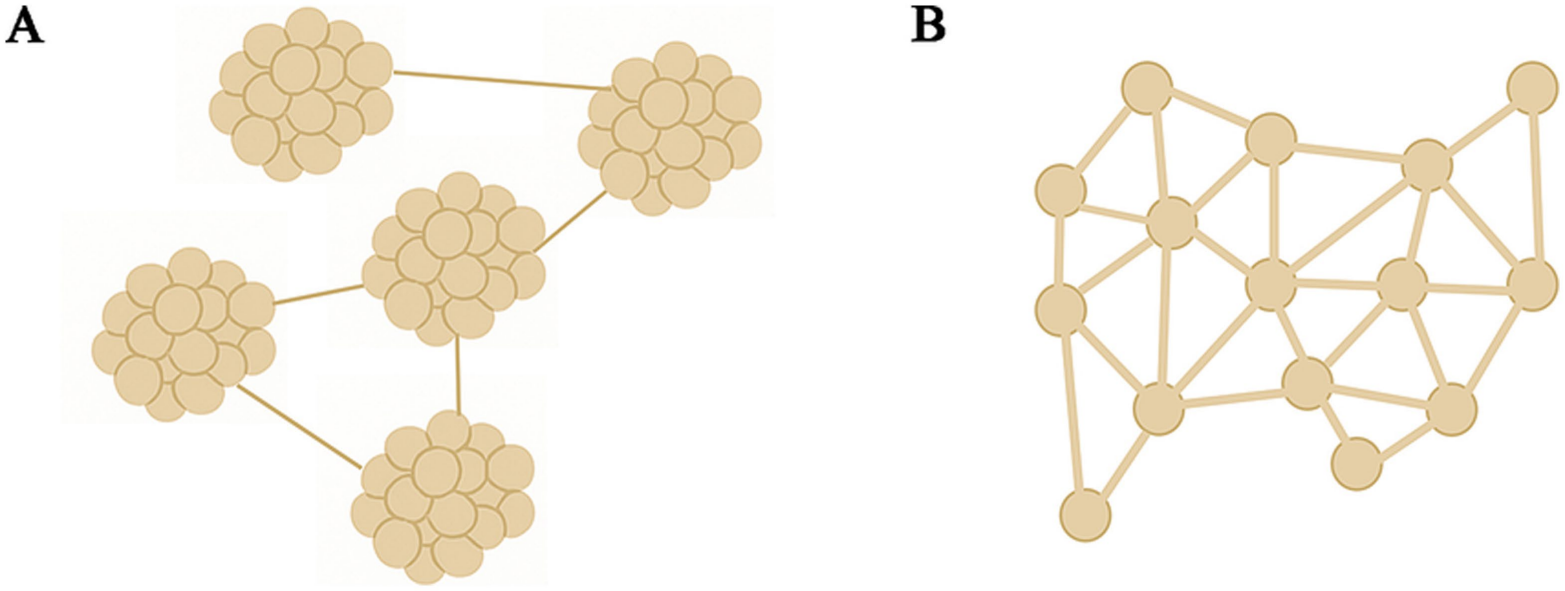
Schematic diagram of the mechanisms by which different water types affect gel network structure; gel network formed under the influence of exogenous divalent cations, e.g., MPC (BTW) gel **(A)**; normal gel network formed under lower divalent cation conditions, e.g., MPC (ROW) gel or MPC (UPW) gel **(B)**.

Microstructural observations (Figure 5) revealed that all MPC gels formed relatively homogeneous three-dimensional network structures. The MPC (BTW) gel exhibited a finer network skeleton with smaller pores, whereas MPC (UPW) gel and MPC (ROW) gel exhibited larger pores and thicker network strands. Previous studies have demonstrated that denser protein networks typically exhibit stronger WHC. This is partly due to the larger contact area between the protein network and water, and partly because smaller pores generate higher capillary pressure, which promotes water retention. In contrast, more open and coarser networks tend to contain larger channels through which water can be expelled more readily under gravity or external force (43, 44). Interestingly, these structural observations appear inconsistent with the WHC results in Figure 4. A possible explanation is that the higher concentrations of divalent cations in boiled tap water weaken electrostatic repulsion and promote salt-bridge formation, causing protein molecules to pre-aggregate during the early stages of gelation, as illustrated in Figure 6. This pre-aggregation generates large protein clusters that do not effectively reorganize into a continuous, uniform three-dimensional network through disulfide bonding or hydrophobic interactions. Consequently, although the microstructure of MPC (BTW) gel shows locally dense regions with fine strands and small pores, these features likely represent intra-cluster compactness rather than effective large-scale network continuity. Such local compactness does not improve WHC; instead, insufficient inter-cluster connectivity restricts the formation of functional pore structures capable of retaining water. The lower final G’ of the MPC (BTW) gel further supports this hypothesis, indicating that pre-aggregation reduced the number of bonds formed between clusters and weakened the elastic response of the resulting gel network. Such pre-aggregation was not observed in the particle size distribution results (3.2.3), which may be attributed to strong electrostatic repulsion maintaining protein dispersion. At neutral pH, the MPC dispersion was far from the isoelectric point of the proteins, thereby effectively preventing pre-aggregation. However, during acidification, the progressive reduction in electrostatic repulsion shortened the distances among proteins. The higher concentrations of divalent cations in boiled tap water, is expected to promote ion-mediated pre-aggregation due to salt-bridge.

#### 3.4.3 Lissajous curves

During processing, storage, transportation, and consumption, gels are often subjected to large deformations and strong shear forces. Under such conditions, nonlinear phenomena may occur, such as yielding, strain stiffening, and structural breakdown, influencing the stability, fracture resistance, and sensory attributes of the material (45). However, parameters such as G’ and G” obtained within the linear viscoelastic region merely reflect the material’s response under slight perturbation. To characterize the nonlinear rheological behavior of the gels after refrigerated storage, LAOS tests were performed. Lissajous curves were therefore used to visualize these nonlinear responses and to illustrate the breakdown of the gel network under large oscillatory deformation.

**Figure 7:**
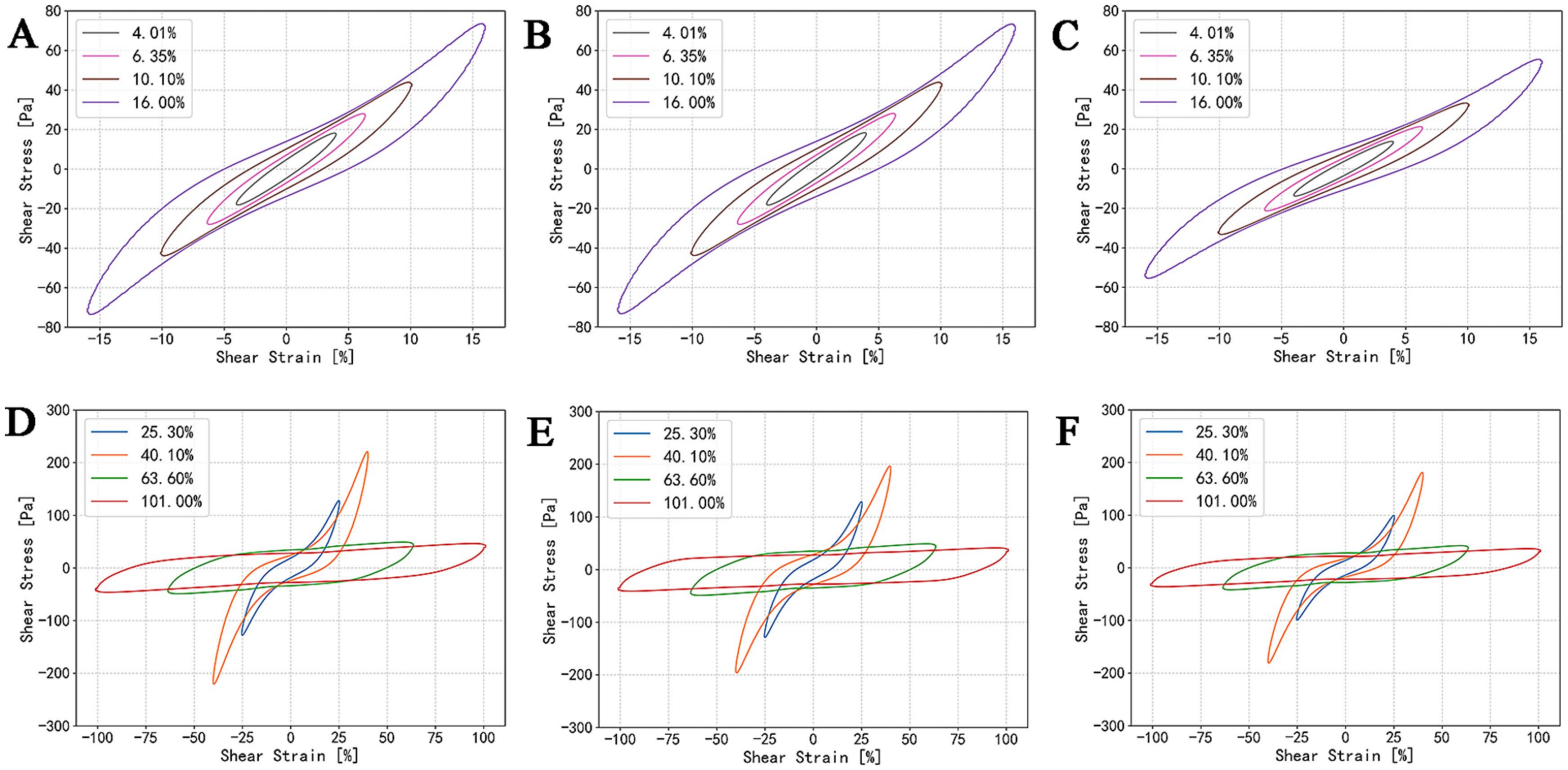
Lissajous curves of MPC gels prepared with ultrapure water **(A,D)**, RO water **(B,E)**, and boiled tap water **(C,F)** under different strain amplitudes; strain amplitude ( $\gamma_{A}$ ) increasing from 4.01 to 16.00%, showing the transition from the linear viscoelastic region (LVE) to the nonlinear viscoelastic region **(A–C)**; strain amplitude ( $\gamma_{A}$ ) increasing from 25.30 to 101.00% **(D–F)**.

As shown in Figures 7A–C, the MPC gels exhibited elliptical Lissajous curves and had comparable stress values at small strain amplitudes ( $\gamma_{A}$ ≤ 6.35%), indicating that the gels remained within the linear viscoelastic region and their stress responses were predominantly elastic. Moreover, in the liner regime, the gels have comparable resistance to deformation. When $\gamma_{A}$ increased to 10.10%, the loops of all samples began to lose their elliptical symmetry, demonstrating the onset of nonlinear viscoelastic behavior. A rapid increase in stress was observed near the strain extrema, indicating strain stiffening, which became more pronounced at $\gamma_{A}=40.10\%$ (Figures 7D–F). When $\gamma_{A}$ reached 63.60%, the stress no longer increased with strain, and the loops transitioned toward a parallelogram-like shape. This transition reflects substantial disruption of the elastic network and the occurrence of yielding during deformation (46). Further increases in strain amplitude did not result in higher peak stresses ( ${\sigma |}_{\gamma =\gamma_{A}}$ ), confirming that the network structure had been severely compromised.

The LAOS results show that, except for the lower stress response observed in the gel prepared with boiled tap water, the maximum linear strain limit and maximum yielding strain were similar among the three samples. This suggests that differences in ionic content among the water samples had no significant effect on the final gel’s resistance to deformation or its brittleness.

## 4 Conclusion

This study compared the gelation properties of MPC gels prepared with food industrial water (RO water) and household water (boiled tap water). The results showed that boiled tap water contained significantly higher concentration of divalent cations than RO water. Although the divalent cation concentration in boiled tap water was relatively low (1.4 mmol/L), this difference was sufficient to affect the structure and properties of MPC gels. Despite having a denser microstructure, gels made with boiled tap water showed lower G′ values and lower water holding capacity compared with those prepared with RO water. This is likely because the divalent cations in boiled tap water promoted pre-aggregation of proteins via electrostatic screening and salt-bridge formation, but insufficient effective cross-linking occurred among aggregates, resulting in weaker gel elasticity. However, because the overall ion content in both water sources remained low, the gels’ appearance, resistance to deformation, and brittleness were not significantly affected, indicating a limited impact on their rheological behavior under large deformation.

It should be noted that the ionic composition of tap water varies across regions depending on water sources and treatment practices. Accordingly, the present results are intended to elucidate general mechanistic trends regarding the role of naturally occurring divalent cations in MPC gelation, rather than to define universally applicable quantitative thresholds. The magnitude of the observed effects may therefore differ with regional water chemistry.

## Data Availability

The raw data supporting the conclusions of this article will be made available by the authors, without undue reservation.
